# To Correspondents

**Published:** 1867-03

**Authors:** 


*To Correspondents.*—A large number of communications are on file, for publication as soon as time permits selection. Several, having reference to meetings of local societies, should have appeared earlier; but, having been accidentally mislaid in the transfer of the "portable property" of the JOURNAL, must be temporarily deferred. In this connection the Editor specially requests secretaries, and others, transmitting proceedings to study brevity and compactness of statement. It is the desire of the JOURNAL to properly notice the transactions of these societies, but it is clearly impossible to print in its pages an entire transcript of their doings. Such papers, treating on professional topics, as the societies may judge valuable and worthy of publication, will be thankfully received and published, if possible; but to secure this publication it must be understood that such papers are to be placed *first* in the possession of this journal. The JOURNAL does not propose to violate the journalistic commandment: *Thou shalt not publish stale news!*

A medical paper should, in the Editor's opinion, aim to give to its readers the gist of the matter—the concentrated essence; neither the crude material, requiring evaporation, or decoction, or distilling, nor the homœopathic dilution. The tendency of all knowledge now is to condensation to pithy aphorisms, and not to expand into unreadable quartos. *Verbum sapientibus.*

